# Rapid screening of *in cellulo* grown protein crystals via a small-angle X-ray scattering/X-ray powder diffraction synergistic approach

**DOI:** 10.1107/S1600576720010687

**Published:** 2020-09-25

**Authors:** Janine Mia Lahey-Rudolph, Robert Schönherr, Cy M. Jeffries, Clément E. Blanchet, Juliane Boger, Ana Sofia Ferreira Ramos, Winnie Maria Riekehr, Dimitris-Panagiotis Triandafillidis, Alexandros Valmas, Irene Margiolaki, Dmitri Svergun, Lars Redecke

**Affiliations:** a Institute of Biochemistry, University of Lübeck, Ratzeburger Allee 160, Lübeck 23562, Germany; b Center for Free-Electron Laser Science (CFEL), Deutsches Elektronen Synchrotron (DESY), Notkestrasse 85, Hamburg 22607, Germany; c Photon Science, Deutsches Elektronen Synchrotron (DESY), Notkestrasse 85, Hamburg 22607, Germany; d European Molecular Biology Laboratory (EMBL), Hamburg Outstation, c/o DESY, Notkestrasse 85, Hamburg 22607, Germany; e Department of Biology, Section of Genetics, Cell Biology and Development, University of Patras, Patras GR-26500, Greece

**Keywords:** *in cellulo* crystals, protein micro-crystallography, small-angle X-ray scattering, X-ray powder diffraction

## Abstract

Crystallization of recombinant proteins in living cells is an exciting new approach for structural biology that provides an alternative to the time-consuming optimization of protein purification and extensive crystal screening steps. Exploiting the potential of this approach requires a more detailed understanding of the cellular processes involved and versatile screening strategies for crystals in a cell culture. Particularly if the target protein forms crystalline structures of unknown morphology only in a small fraction of cells, their detection by applying standard visualization techniques can be time consuming and difficult owing to the environmental challenges imposed by the living cells. In this study, a high-brilliance and low-background bioSAXS beamline is employed for rapid and sensitive detection of protein microcrystals grown within insect cells. On the basis of the presence of Bragg peaks in the recorded small-angle X-ray scattering profiles, it is possible to assess within seconds whether a cell culture contains microcrystals, even in a small percentage of cells. Since such information cannot be obtained by other established detection methods in this time frame, this screening approach has the potential to overcome one of the bottlenecks of intracellular crystal detection. Moreover, the association of the Bragg peak positions in the scattering curves with the unit-cell composition of the protein crystals raises the possibility of investigating the impact of environmental conditions on the crystal structure of the intracellular protein crystals. This information provides valuable insights helping to further understand the *in cellulo* crystallization process.

## Abstract 2

A rapid and sensitive detection approach utilizing high-brilliance and low-background small-angle X-ray scattering and X-ray powder diffraction to detect protein microcrystals grown within living insect cells is described.

## 1. Introduction   

Nowadays, it is well established that living cells from all kingdoms of life possess an intrinsic ability to form intracellular protein crystals, denoted as ‘*in vivo* grown crystals’ or ‘*in cellulo* crystals’ (Schönherr *et al.*, 2018 ▸). The assembly of intracellular proteins into native crystalline states could provide specific advantages for the organism, mainly in terms of storage and protection. However, this phenomenon also applies to recombinant proteins produced by heterologous gene expression, as highlighted by the growing number of examples predominantly observed in mammalian and baculovirus-infected insect cells. During recent years, novel developments in serial crystallography data collection strategies on X-ray free-electron lasers (XFELs) and synchrotron sources (Standfuss & Spence, 2017 ▸; Yamamoto *et al.*, 2017 ▸; Yabashi & Tanaka, 2017 ▸) have paved the way to use *in cellulo* crystals with dimensions in the low micrometre or even the nanometre size range as suitable targets for X-ray crystallography (Gati *et al.*, 2014 ▸; Schönherr *et al.*, 2018 ▸). High-resolution structural information on several recombinant proteins has already been obtained from diffraction of *in cellulo* crystals, *e.g.* for the coral *Dipsastraea favus* derived fluorescent protein Xpa (Tsutsui *et al.*, 2015 ▸), the metazoan-specific human kinase PAK4 in complex with Inka1 (Baskaran *et al.*, 2015 ▸) and the BinAB larvicide from *Lysinibacillus sphaericus* (Colletier *et al.*, 2016 ▸), as well as of cathepsin B (CatB; Redecke *et al.*, 2013 ▸) and IMP de­hydrogenase (IMPDH; Nass *et al.*, 2020 ▸) from the parasite *Trypanosoma brucei*.

These results question the earlier opinion that the crowded environment in living cells might impact the order of the crystalline structure (Doye & Poon, 2006 ▸). Moreover, they indicate that *in cellulo* protein crystallization is able to offer exciting possibilities complementary to conventional crystallization techniques (Chayen & Saridakis, 2008 ▸). The approach is particularly important for proteins that were/are not accessible for crystallization using established *in vitro* screening strategies, as shown for *T. brucei* IMPDH (Nass *et al.*, 2020 ▸) and fully glycosylated *T. brucei* CatB (Redecke *et al.*, 2013 ▸). *In cellulo* crystallization provides an alternative to the time-consuming optimization of protein purification and extensive crystal screening steps. Additionally, the quasi-native conditions in host cells prevent crystal distortion that could arise from non-physiological conditions imposed by re-crystallization and provide the opportunity to identify native co-factors present in the highly versatile natural reservoir of compounds within living cells (Nass *et al.*, 2020 ▸). However, exploiting the tremendous potential of *in cellulo* protein crystallization requires a more detailed understanding of the cellular processes involved in crystal formation. Insights into the mechanisms that control the size and shape of crystals, and also the identification of biological parameters suitable for screening approaches, could further widen the applications of *in cellulo* crystallization.

On the basis of a detailed comparison of reported intracellular protein crystallization events, specific requirements have been proposed to favour *in cellulo* crystal growth in fruitful interplay (Koopmann *et al.*, 2012 ▸; Schönherr *et al.*, 2015 ▸, 2018 ▸; Duszenko *et al.*, 2015 ▸). This includes the intrinsic crystallization tendency of the target protein under the specific environmental conditions provided by the individual cellular compartments. Moreover, high local protein concentrations seem to be required, which might result from a preceding protein phase separation event (Hasegawa, 2019 ▸). In insect cells, crystals occurred in the endoplasmic reticulum (CatB; Koopmann *et al.*, 2012 ▸) and in peroxisomes (IMPDH, luciferase; Nass *et al.*, 2020 ▸; Schönherr *et al.*, 2015 ▸), depending on the native translocation signals harboured in the sequence of the recombinant proteins. Furthermore, a cytosolic localization of crystals was observed [calcineurin, avian reovirus nonstructural protein fused to green fluorescent protein (GFP-μNS), IMPDH; Fan *et al.*, 1996 ▸; Schönherr *et al.*, 2015 ▸; Nass *et al.*, 2020 ▸]. Thus, different cellular environments may represent the basis for developing a more systematic *in cellulo* crystallization screening approach that would exploit living cells as crystallization factories for a large number of recombinant proteins. An initial strategy to test the crystallization capability of living insect cells has already been proposed and applied to recombinant CPV1 polyhedrin crystals (Boudes *et al.*, 2016 ▸, 2017 ▸).

The successful detection of protein crystals inside living cells represents a crucial – and somewhat challenging – task in the development of a versatile screening strategy for *in cellulo* crystallization. During recent years a variety of methods have been optimized to identify even nanometre-sized protein crystals in conventional crystallization setups and to locate these crystals after mounting at the beamline (Becker *et al.*, 2017 ▸). Unfortunately, the environmental challenges imposed by the living cells largely prevent the direct and efficient detection of *in cellulo* crystals. Most frequently, bright-field microscopy methods including contrast enhancement techniques, *e.g*. differential interference contrast (DIC) or integrated modulation contrast, are applied to visualize the intracellular crystals (Schönherr *et al.*, 2015 ▸). The main advantages of these non-invasive methods include frequently accessible equipment, the lack of elaborate sample preparation steps and the good visualization of sufficiently sized crystals. However, the limited resolution of visible-light-based approaches combined with marginal differences in refractive indices makes it difficult to reliably differentiate the ordered crystalline structures in the nanometre size range from the chaotic cellular background. For nanocrystals, transmission electron microscopy (TEM) (Stevenson *et al.*, 2014 ▸) was developed into a tool that enables the study and optimization of crystal formation processes *in vitro* (Stevenson *et al.*, 2016 ▸) and can be used to characterize *in cellulo* crystals directly within the cellular environment. A resolution in the low nanometre size range allows the visualization of the crystal structure, which can also be applied to identify *in cellulo* crystals (Schönherr *et al.*, 2018 ▸). However, since TEM requires ultrathin sectioning (usually <90 nm), a crystal-containing cell has to be selected by chance from the entire population and the crystal must be intersected by the ultrathin cut. If intracellular crystal growth is restricted to a few cells in the entire culture or only very few nanocrystals per cell are produced, this represents a significant limitation, which, together with the time-consuming sample preparation, hampers the simple and rapid detection of crystals in a cell culture. Second harmonic generation (SHG) microscopy used in combination with UV two-photon excited fluorescence, and frequently referred to as second-order nonlinear optical imaging of chiral crystals (SONICC; Kissick *et al.*, 2011 ▸; Haupert *et al.*, 2012 ▸), represents another emerging technique to rapidly achieve successful crystal formation in conventional screening setups with high sensitivity, selectivity and the potential for automatization (Becker *et al.*, 2017 ▸; Tang *et al.*, 2020 ▸). However, UV fluorescence is less helpful for intracellular crystals owing to the high protein concentration surrounding the crystal in the cellular environment, and high crystal symmetry may reduce the crystal-specific SHG signal in practice by about two orders of magnitude (Haupert *et al.*, 2012 ▸). Together with the possibility of SHG signal generation by filaments within the cells (Campagnola & Loew, 2003 ▸) this could prevent a reliable *in cellulo* crystal detection.

A direct proof for the presence of crystallites is given by the detection of specific Bragg diffraction of electrons or X-rays from a sample. The technique of micro-electron diffraction has the potential to unravel structures of proteins and other biological molecules at 1–3 Å resolution from a few crystals in the nanometre size range, because of the strong interaction between electrons and the crystal. However, ultrathin samples are required, which are frequently obtained by milling (Shi *et al.*, 2013 ▸; Jones *et al.*, 2018 ▸). X-ray powder diffraction (XRPD) provides a fingerprint of every crystalline phase exhibiting a unique diffraction pattern, and differences between the various crystalline forms can be observed by examining the peak positions and intensities in XRPD patterns (Katrincic *et al.*, 2009 ▸). Even small changes in the form of new peaks, additional shoulders or shifts in the peak positions often imply the presence of a second polymorph (Davidovich *et al.*, 2004 ▸). Thus, information about crystalline sample composition is obtained, yielding knowledge of whether the sample consists of one or more phases. During the past decade, XRPD has moved beyond fingerprinting of microcrystalline samples by extraction of accurate lattice parameters, elucidating new structural information from biological macromolecules at low and medium resolution (Von Dreele, 2019 ▸; Karavassilia & Margiolaki, 2016 ▸; Karavassili *et al.*, 2017 ▸; Spiliopoulou *et al.*, 2020 ▸; Margiolaki, 2019 ▸). Densely packed, randomly oriented crystals produce Debye–Scherrer rings on the detector that allow the evaluation of the diffraction capabilities of the sample (Von Dreele *et al.*, 2000 ▸; Margiolaki *et al.*, 2007 ▸). Even if a relatively small number (<50) of low-angle peaks is considered to be sufficient to precisely refine the unit-cell parameters (Von Dreele, 2019 ▸), the volume of the cellular soft matter that surrounds intracellular crystals significantly restricts the crystal density. Thus, the powder diffraction intensity of intracellular crystals at synchrotron crystallography beamlines is often restricted, especially when the crystal-to-cell number ratio in the sample is low (Margiolaki & Wright, 2008 ▸).

Small-angle X-ray scattering (SAXS) is performed in solution to structurally characterize biological macromolecules under dilute conditions. SAXS instruments are optimized to minimize the scattering background to detect weak scattering signals that are often orders of magnitude smaller in intensity than diffraction peaks. SAXS profiles provide information on size, shape and oligomerization state but also about interactions between particles in solution. SAXS is extremely sensitive to the formation of crystallites, and this technique has previously been used to analyse protein nucleation (Kovalchuk *et al.*, 2016 ▸) and crystallization kinetics (Poplewska *et al.*, 2019 ▸). Furthermore, the micro- and nano-GISAX method could even significantly exceed the sensitivity of the SAXS technique for studying protein nucleation (Pechkova & Nicolini, 2017 ▸).

In this study, we exploited SAXS and XRPD for a rapid and sensitive detection of protein microcrystals grown within insect cells. We employed the high-brilliance and low-background P12 bioSAXS beamline (Blanchet *et al.*, 2015 ▸) of the EMBL at the PETRA III storage ring (DESY, Hamburg). Four test proteins were measured: *Photinus pyralis* luciferas, *T. brucei* IMPDH and CatB, and *Neurospora crassa* HEX-1. Mock-virus-infected and uninfected cells were used as a control. Combining the high sensitivity of SAXS with XRPD analysis methods, we demonstrate that it is possible to assess within seconds whether a cell culture contains microcrystalline material based on the presence of Bragg peaks in the recorded scattering profiles, even for target proteins that form crystals only in a small percentage of cells. This screening approach has the potential to overcome the methodological bottleneck of crystal detection within living cells and opens up opportunities to investigate and understand the influence of growth conditions, stress, temperature, starvation, cellular compartmentalization and the choice of cell line on the size and formation of *in cellulo* crystals.

## 2. Methods   

### 2.1. Cloning   

Cloning procedures for *T. brucei* IMPDH (gene bank accession number M97794) and *T. brucei* CatB (gene bank accession number AY508515) have been described previously (Nass *et al.*, 2020 ▸; Koopmann *et al.*, 2012 ▸). The genes coding for *P. pyralis* luciferase (Luc, gene bank accession number AB644228) and *N. crassa* HEX-1 (gene bank accession number XM_958614) were amplified by PCR using primers 5′-GAAGACGCCAAAAACATAAAGAA-′3 (sense) and 5-CAATTTGGACTTTCCGCCCTTC-3′ (antisense), and 5′-TACTACGACGACGACGCTCACG-′3 (sense) and 5′-GAGGCGGGAACCGTGGACG-3′ (antisense), respectively. ALLin HiFi DNA polymerase (highQu) was used according to the manufacturer’s instructions. The amplicons were ligated into a modified pFastBac1 vector (Thermo Scientific) containing the sequence 5′-ATGGGCGCCTAA-3′ between the *Bam*HI and *Hind*III restriction sites to accommodate an *Ehe*I restriction site. The vector was linearized using FastDigest *Ehe*I (Thermo Scientific) and blunt-end ligation was achieved using T4 DNA ligase (Thermo Scientific) according to the manufacturer’s protocol. Plasmids were transformed into competent *Escherichia coli* DH5α cells (Stratagene) and purified (GeneJET plasmid miniprep kit, Thermo Scientific). The integrity of the cloned sequences was verified by Sanger sequencing. All generated pFastBac1 plasmids were transformed into competent *E. coli* DH10EmBacY cells (Geneva Biotech) according to the manufacturer’s instructions. Recombinant bacmid DNA was purified using the GeneJET plasmid miniprep kit (Thermo Scientific) and subsequently used for PCR analysis of the transposed sequence, employing standard pUC M13 forward and reverse primers. For mock-virus generation, bacmid DNA was directly isolated from *E. coli* DH10EmBacY cells without prior transposition of a recombinant gene of interest.

### 2.2. Insect cell culture   

Sf9 and High Five insect cells were held in suspension culture in serum-free ESF921 insect cell culture medium (Expression Systems) at 300 K on an orbital shaker at 100 r min^−1^. Suspension culture cells were seeded at 0.5–1 × 10^6^ cells ml^−1^, in a total volume of 25 ml in an upright-standing 75 cm^2^ disposable T-flask. Cell density was counted daily and cultures were split when the density reached 4 × 10^6^ cells ml^−1^ for High Five or 6 × 10^6^ cells ml^−1^ for Sf9 cells.

### 2.3. Recombinant virus generation   

Recombinant bacmid DNA was used for lipofection with Sf9 insect cells grown in ESF921 serum-free medium at 300 K using Escort IV reagent (Sigma–Aldrich) according to the manufacturer’s instructions. In brief, 0.45 × 10^6^ Sf9 cells per well in a 12-well plate were transfected with 1 µg of bacmid DNA and 3 µl of Escort IV reagent for 18 h. After 4 days of incubation at 300 K the first supernatant (P1) was harvested by centrifugation at 21 000 relative centrifugal force (r.c.f.) for 30 s. For high-titre stock production (third passage, P3), 0.9 × 10^6^ Sf9 cells per well in a six-well plate were infected with 100 µl of P1 or 20 µl of P2 viral stock and incubated for 4 days. Viral P2 and P3 stocks were harvested as described above.

### 2.4. Viral titre determination   

A serial dilution assay was used to calculate the titre of the viral P3 stocks. In a 96-well plate, a suspension of 3 × 10^4^ High Five cells in 180 µl of antibiotic-free ESF921 insect cell culture medium was added to each well and incubated for 30 min to let cells attach to the bottom. Then, a 1:10 dilution of the virus solution with medium was prepared and 20 µl portions of this solution were added to each of six wells of the first row. For each serial dilution step the medium containing the virus was mixed in the well using a multi pipette and 20 µl of the supernatant was transferred into the next row. Pipette tips were discarded after each row; eight rows were prepared per titration. After 4 days at 300 K, enhanced yellow fluorescent protein (EYFP) fluorescence indicating a successful infection was evaluated, and wells with at least two fluorescent cells were counted as positive. The virus titre was calculated using the TCID_50_ (tissue culture infectious dose; Reed & Muench, 1938 ▸).

### 2.5. Sample preparation for X-ray measurements   

In one well of a six-well cell culture plate, 8 × 10^5^ Sf9 or High Five cells were plated in 2 ml of ESF921 insect cell culture medium and subsequently infected with P3 stock of the recombinant baculovirus (rBV) using a multiplicity of infection (MOI) of 1. Cells were incubated as a semi-adherent culture at 300 K for 40–96 h until needed for the diffraction experiments. The cells were then gently flushed from the well bottom with a 1000 µl pipette and centrifuged for 30 s at 270 r.c.f., and the cell pellet was resuspended in 25 µl of Tris-buffered saline (TBS; 20 m*M* Tris, 150 m*M* NaCl pH 7.0). 40–45 µl of this suspension was transferred into the sample tubes and immediately used for the X-ray scattering experiments.

For dilution series of crystal-carrying cells, High Five insect cells expressing the target gene were mixed with mock-rBV-infected cells in a 1:2 ratio. Up to seven serial dilution steps were carried out directly prior to the X-ray scattering experiments with samples prepared in TBS as previously mentioned.

### 2.6. Light microscopy   

For cell and crystal counting, cell cultures were imaged with a Leica DM IL LED microscope equipped with a 20× objective and a Leica MC170 HD microscope camera prior to the diffraction experiment. The crystal-containing cells and those without crystals were manually counted, and their ratio was calculated. The images of the cell cultures were generated using a Zeiss Observer.Z1 inverted microscope with a 20× objective and an AxioCam MRm microscope camera.

### 2.7. Propidium iodide staining of infected cells   

To visualize the effects of the sample preparation procedure on the cell viability, High Five insect cells were infected as described above for diffraction experiments. Four days after infection, cells were imaged within the wells on a Zeiss Observer.Z1 microscope using differential interference contrast mode and wide field fluorescence. The cells were then gently flushed from the well bottom with a 1000 µl pipette and centrifuged for 30 s at 270 r.c.f., and the cell pellet was resuspended in 25 µl of TBS containing 500 ng ml^−1^ of propidium iodide. Cells were incubated for 10 min at room temperature and then spread on a glass coverslip and imaged again as described above. All samples were prepared in triplets, imaged and manually counted.

### 2.8. X-ray data collection   

Data were collected at the EMBL P12 beamline (PETRA III, DESY, Hamburg, Germany) (Blanchet *et al.*, 2015 ▸). A photon energy of 10 keV (1.24 Å) was used throughout the experiments, with a photon flux of about 10^13^ ph s^−1^ at the sample position. Data [*I*(*s*) versus *s*, where *s* = 4πsin(θ)/λ, 2θ is the scattering angle and λ is the X-ray wavelength] were recorded at a sample–detector distance of 3.00 m using a Pilatus 6M detector (setup 1) or a Pilatus 2M detector (setup 2), both from DECTRIS, Switzerland. 40–45 µl of the insect cell suspension prepared as described above was loaded bubble free into the reaction vessels of the SAXS setup, of which 30 µl was transferred into a temperature-controlled 1.8 mm quartz capillary using the automatic bioSAXS sample changer (Arinax) (Round *et al.*, 2015 ▸). The high cell density prevented cell settling in the sample tube during the automated loading of up to eight consecutive samples by the sample changer robot.

Using a focal spot of 0.2 × 0.12 mm (FWHM) in a fixed-flow measurement at 293 K, 40 detector frames were recorded per sample separated by 40 buffer frames, all with a single-frame exposure time of 0.045 s and a readout time of 0.005 s, resulting in a total exposure time of 4 s per data set. For each cell sample, a single data set was collected with the corresponding buffer (TBS), enabling the buffer subtraction during data analysis.

### 2.9. Data processing   

For each sample and corresponding buffer measurement, the 40 individual 2D-detector data frames collected during the course of exposure were summed to produce a final 2D image that was subsequently radially averaged using *im2dat* (Franke *et al.*, 2017 ▸) to generate 1D scattering profiles (data deposited with the Small Angle Scattering Biological Data Bank, SASBDB; http://www.sasbdb.org). The data measured from the TBS control were then subtracted, applying the *ATSAS* program suite (Petoukhov *et al.*, 2012 ▸; ). 1D profile plots were created with *PRIMUS* (Konarev *et al.*, 2003 ▸). The data were converted from *I*(*s*) to *I*(2θ) to facilitate indexing and profile refinement with software packages designed for the analysis of XRPD data as described in the following sections.

### 2.10. Data clustering and Pawley analysis   

Since indexing of acquired data was not feasible owing to the paucity of diffraction peaks, information about data similarities has been evaluated via principal component analysis (PCA) on the *I*(2θ) data of all data sets over the 0.4–2.0° 2θ range, using *HighScore Plus* (Degen *et al.*, 2014 ▸). This program was also used to extract accurate unit-cell parameters by applying the Pawley approach (Pawley, 1981 ▸) for whole powder pattern fitting (WPPF). In the absence of indexing solutions, reasonable starting values for unit-cell parameters were retrieved from relevant PDB entries (Supplementary Table S1). Peak profiles were simulated using a pseudo-Voigt function with the standard description for FWHM and peak asymmetry variation over the 2θ range (Von Dreele, 2019 ▸). The background was initially estimated and later it was modelled after a shifted Chebyshev polynomial with varying number of terms (∼10–14), depending on the data set, which were refined during Pawley analysis. Parameters were included for refinement of the polynomial background, as well as for instrumental angular offset (zero shift). In the case of highly overlapping reflections, the intensity was equipartitioned to the constituent peaks and gradually refined.

## 3. Results and discussion   

Four test proteins were measured to evaluate the capability of the low-background SAXS beamline P12 for a reliable intracellular crystal detection in living insect cells. Of these proteins, three are known to crystallize in living insect cells infected by rBV, but they differ in crystallization efficiency, as well as in crystal volume and morphology. *T. brucei* IMPDH and CatB have previously been reported to form micrometre-sized needle-shaped crystals in most cells from populations that diffract XFEL pulses and synchrotron radiation to high resolution, enabling the elucidation of the corresponding protein structures (Koopmann *et al.*, 2012 ▸; Redecke *et al.*, 2013 ▸; Gati *et al.*, 2014 ▸; Nass *et al.*, 2020 ▸). Needle-shaped *in cellulo* crystals were also observed for firefly (*P. pyralis*) luciferase, growing up to a remarkable length of more than 180 µm, but the spontaneous disintegration after cell membrane disruption has prevented the validation of X-ray diffraction so far (Schönherr *et al.*, 2015 ▸). Additionally, HEX-1, a natively self-assembling protein that forms the solid, crystalline core of Woronin bodies in the fungus *N. crassa* (Tenney *et al.*, 2000 ▸), assembles into regular spindle-shaped crystals with a hexagonal cross section in almost all insect cells of the culture, which has not been reported previously.

### 3.1. Detection of *in cellulo* crystals using SAXS and XRPD   

**Figure 1:**
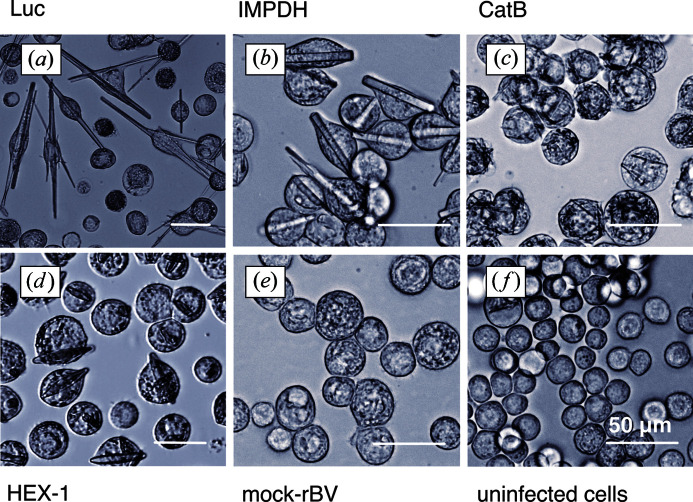
Detection of protein crystals in living insect cells. Light-microscopic images of High Five insect cells 4 days after infection with recombinant baculoviruses (MOI = 1) encoding (*a*) *P. pyralis* Luc, (*b*) *T. brucei* IMPDH, (*c*) *T. brucei* CatB and (*d*) *N. crassa* HEX-1. (*e*) Mock-rBV-infected cells; (*f*) uninfected cells. The intracellular crystals of the individual test proteins differ in size and morphology.

**Figure 2:**
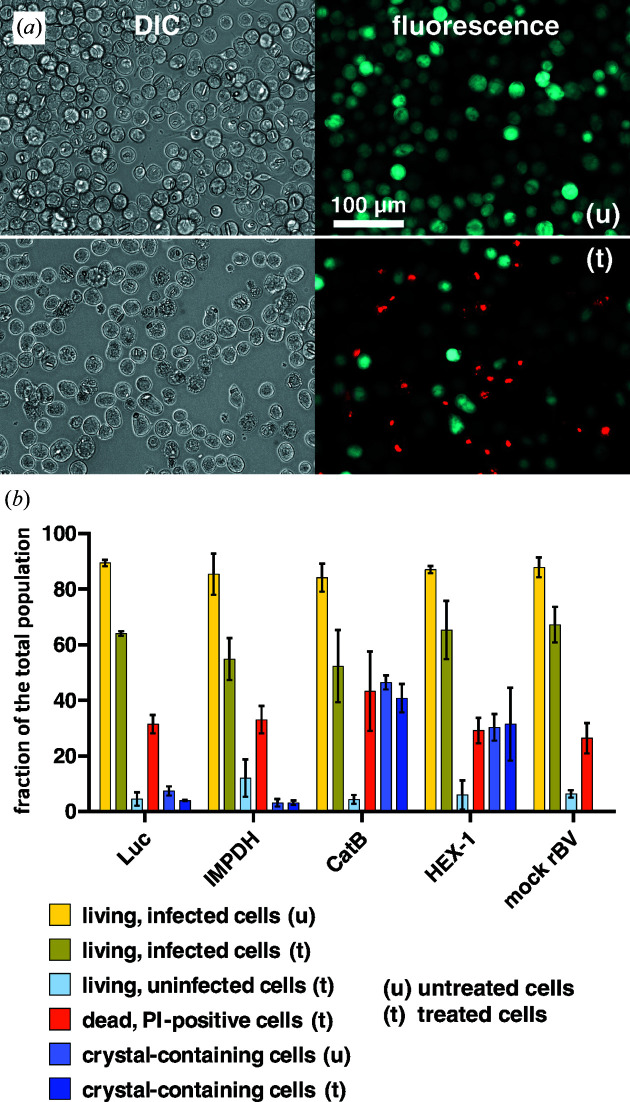
Effects of sample preparation procedures on the viability of infected High Five insect cells. (*a*) High Five cells imaged 4 days after infection with rBV HEX-1. Upper panel: differential interference contrast light microscopy and fluorescence microscopy of insect cells prior to sample preparation (u, untreated cells). EYFP fluorescence labelling of living, baculovirus-infected cells is shown in cyan. Lower panel: DIC and fluorescence microscopy of cells after sample preparation and propidium iodide (PI) staining (t, treated cells). PI fluorescence labelling of dead cells is shown in red. The scale bar applies to all panels. (*b*) Analysis of fractions of living, dead, uninfected and crystal-containing cells prior to and after sample preparation procedures within different High Five cell cultures. More than 60% of rBV-infected High Five cells are still vital after sample preparation and thus at the beginning of the diffraction experiment. The reduction of luciferase-crystal-containing cells is due to the instability of luciferase crystals outside the living cell.

Prior to the diffraction experiment, the previously observed intracellular crystallization tendency of the test proteins in rBV-infected High Five insect cells was verified by light microscopy at day 4 post infection (p.i.). No ordered structures have been detected in the uninfected and in the mock-rBV-infected cells, which served as controls for the subsequent diffraction experiments. The percentage of crystal-containing cells within the entire culture, subsequently denoted as ‘crystallization efficiency’, was estimated to be around 70–80% for Luc, 40–60% for IMPDH, 50–90% for CatB and more than 90% for HEX-1, slightly varying depending on the individual culture (Fig. 1 ▸). Immediately before X-ray experiments at P12, dense cell suspensions were prepared in TBS. At this stage, 60–80% of the rBV-infected cells are still vital in all samples, as confirmed by propidium iodide staining (Fig. 2 ▸). Thus, neither virus infection and intracellular crystal growth nor the sample preparation procedures affected the integrity of the predominant fraction of the High Five cells. Moreover, the percentage of crystal-containing cells remained almost constant during sample preparation. Only for luciferase-producing cells was the proportion of crystal-containing cells significantly reduced, from approximately 50 to 30% [Fig. 2 ▸(*b*)], which can be attributed to the instability of *in cellulo* grown luciferase crystals outside the living cell (Schönherr *et al.*, 2015 ▸).

**Figure 3:**
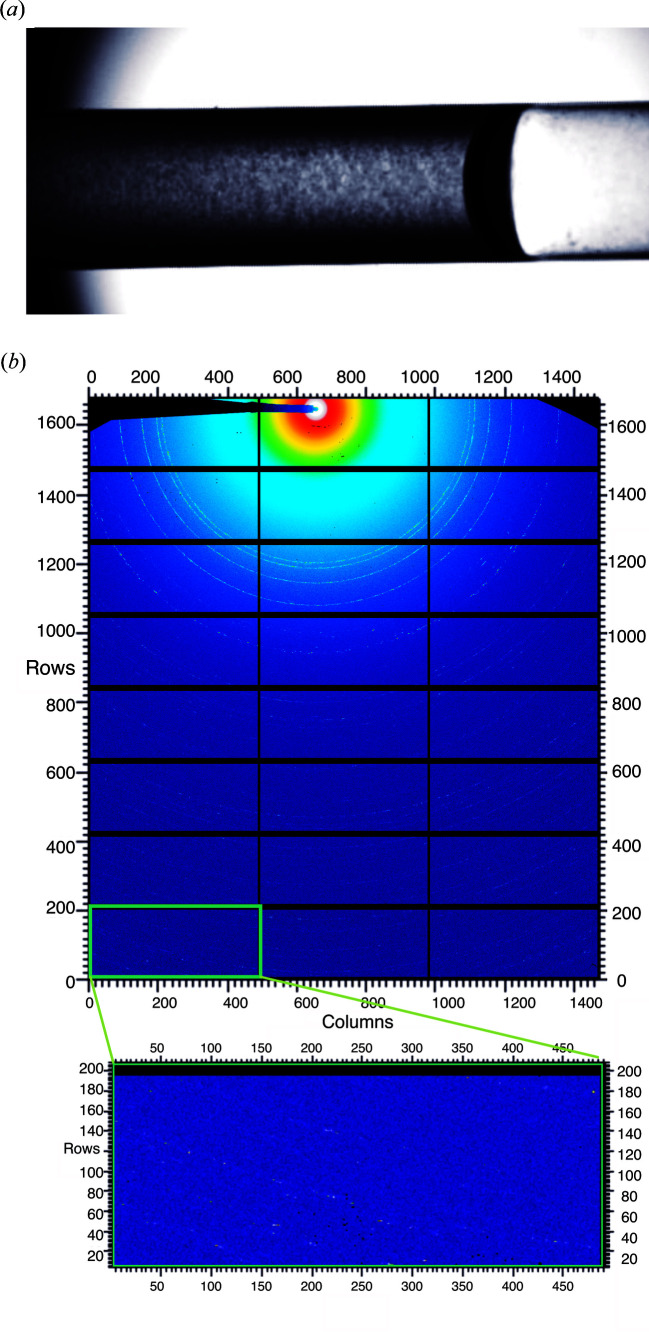
(*a*) Snapshot of the quartz capillary filled with the dense crystal-containing cell suspension during X-ray diffraction data collection. (*b*) Summed detector image (40 frames) of HEX-1 crystals irradiated in living High Five insect cells collected using the Pilatus 2M photon-counting detector (DECTRIS) at the EMBL SAXS beamline P12 (PETRA III, DESY, Hamburg, Germany). Granular Debye–Scherrer rings can be detected up to the edge of the detector image (inset). The image was generated using the program *FIT2D* (Hammersley, 2016 ▸).

The samples were automatically loaded with a robotic sample changer into the quartz capillary for X-ray diffraction [Fig. 3 ▸(*a*)]. The short exposure time of 0.045 s and readout time of 0.005 s per frame in the steady-state mode resulted in a measurement time per sample of 4 s, since 40 detector frames have individually been recorded for each sample, followed by 40 frames of buffer irradiation. Additionally considering the time required for the automated sample loading and removal as well as capillary cleaning, eight consecutive diffraction data sets were collected within 24 min without opening the hutch, representing the optimal agreement between efficient data collection and settling and survival of the insect cells in TBS. If the previously scored crystal-containing cells had been irradiated, summation of the detector frames consistently revealed the presence of Debye–Scherrer rings [Fig. 3 ▸(*b*)] resulting from the orientational average of the Bragg reflections from the many small crystals randomly oriented in the cell suspension, a typical observation during XRPD measurements. No rings were observed for the control samples.

**Figure 4:**
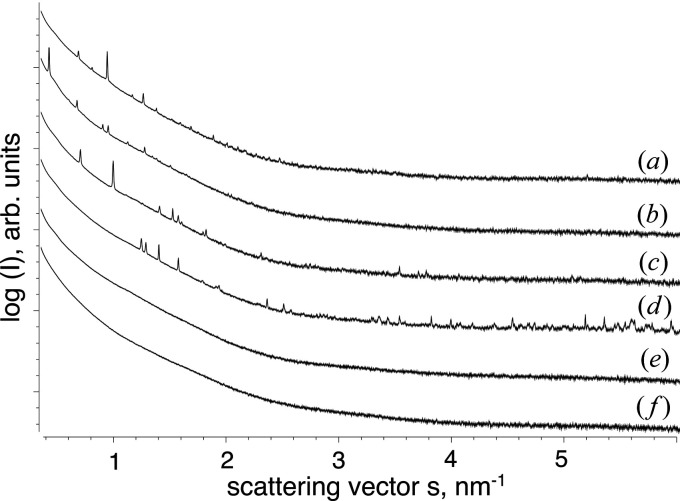
1D radially averaged X-ray scattering data of the insect cells containing intracellular crystals of the target proteins (*a*) Luc (SASBDB: SASDHY5), (*b*) IMPDH (SASBDB: SASDHZ5), (*c*) CatB (SASBDB: SASDH26) and (*d*) HEX-1 (SASBDB: SASDH36). (*e*) Mock-rBV-infected cells (SASBDB: SASDH46); (*f*) uninfected cells (SASBDB: SASDH56). If crystalline structures are present within the cells, distinct Bragg diffraction peaks are detected in the scattering curves at scattering vectors characteristic for the unit-cell dimensions of the protein crystals. All scattering curves were acquired with detector setup 1.

Subtraction of the buffer signal and radial averaging resulted in 1D plots representing the intensity versus momentum transfer *s* (Fig. 4 ▸). The corresponding real-space distances are determined as *d* = 2π/*s*. The scattering curves of the crystal-containing cell samples exhibit clear peaks at defined *s* values, representing the Debeye–Scherrer rings [Fig. 4 ▸, curves (*a*)–(*d*)]. Depending on its unit cell, each crystal type produced a distinct XRPD profile that can act as a fingerprint of the crystallite.

The intensity of the peaks depends on the overall scattering capability of the irradiated part of the sample. Using an X-ray beam of 0.20 × 0.12 mm and a 1.8 mm quartz capillary, a volume of 0.043 mm^3^ is irradiated, which could incorporate several thousand cells, estimating a diameter of approximately 0.030 mm per cell. Comparable magnitudes of the scattering intensity can be recorded in the case when just a few relatively large crystals are present within the irradiated volume, or when a large number of small crystals are illuminated – it is important that the total number of crystallographic unit cells is above the detection limit defined by the photon flux of the X-ray beam. Thus, the comparatively low crystallization efficiency of IMPDH (40–60%) and Luc (70–80%) is compensated by the significantly increased scattering volume of these long needle-shaped crystals (Fig. 1 ▸), resulting in a comparable intensity of the dominant scattering peaks observed for the more abundant but smaller crystals of HEX-1 (>90% efficiency) and CatB (up to 90% efficiency). Consequently, the presence of specific peaks in the scattering curve reliably indicates the presence of crystalline structures with the scattering volume suitable for detection at the given experimental conditions, but the peak intensity on its own does not represent a suitable measure to compare the number and/or size of different crystallites in the living cells (Fig. 3 ▸).

### 3.2. Extraction of refined unit-cell parameters   

The low-angle region of XRPD data usually allows for a precise refinement of the unit-cell parameters of the diffracting crystals, if pure and highly dense microcrystalline suspensions are used in conventional powder diffraction experiments (Margiolaki, 2019 ▸). In our samples, a significant volume is occupied by the solvent and the soft matter of the cells, limiting the accessible crystal density and thus the intensity of the Bragg scattering patterns. Only a few significant peaks at low *s* values can be detected in the scattering curves of intracellular Luc, IMPDH, CatB and HEX-1 crystals (Fig. 4 ▸), preventing *ab initio* indexing.

It has been demonstrated in earlier studies (Norrman *et al.*, 2006 ▸; Fili *et al.*, 2015 ▸; Valmas *et al.*, 2015 ▸) that information about data similarities can be evaluated via PCA. PCA reduces the dimensionality of data sets by projecting them to distinct principal component (PC) axes, which are planes in the multidimensional space (Hotelling, 1933 ▸). By definition, the first PC is the plane where data exhibit the largest variance when projected along it. Subsequent PCs must be orthogonal to the first one. Once the required number of PCs is identified (typically two or three), data are projected into a new coordinate system defined by these PCs. The position of each observation in the PC coordinate system and its distance to other observations is indicative of the similarities between the observations. Analysis performed on the *I*(2θ) data over the 0.4–2.0° 2θ range produced four distinct clusters for the samples under study, each containing one of the four different phases observed in our experiments (Supplementary Fig. S1). Clustering not only allowed us to detect the existence of four well separated crystalline phases in our data (marked A–D in Supplementary Fig. S1), even before their identification, but also enhanced the rapidity of the analysis.

**Figure 5:**
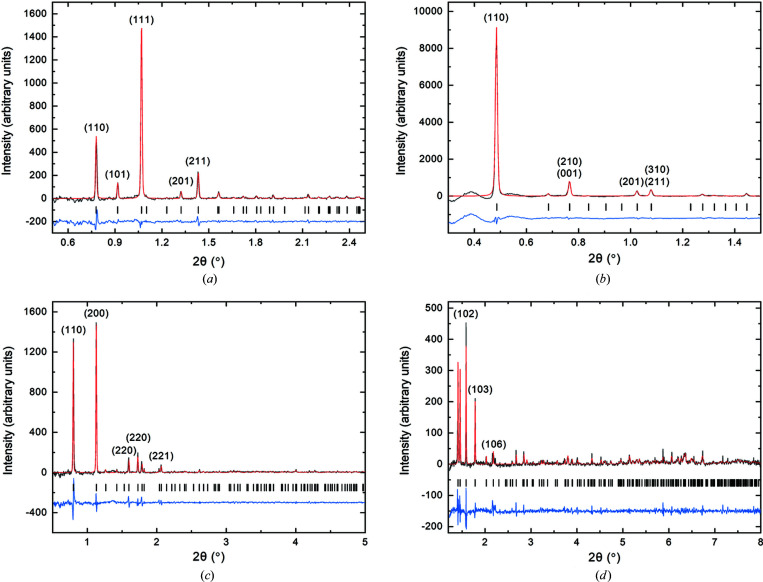
Pawley fits of XRPD data of High Five insect cells containing intracellular crystals of the target proteins (*a*) Luc (SASBDB: SASDHY5), (*b*) IMPDH (SASBDB: SASDHZ5, (*c*) CatB (SASBDB: SASDH26) and (*d*) HEX-1 (SASBDB: SASDH36). Background has been subtracted for clarity. The black, red and blue lines represent the experimental data, the calculated profile, and the difference between experimental and calculated patterns, respectively. A few of the major peaks have been annotated. The vertical black lines correspond to Bragg reflections compatible with the respective refined unit cells: (*a*) *P*4_1_2_1_2, *a* = *b* = 129.13 (6) Å, *c* = 97.1 (1) Å, *R*_wp_ = 0.80, χ^2^ = 1.76, (*b*) *P*42_1_2, *a* = *b* = 209.3 (1) Å, *c* = 93.44 (2) Å, *R*_wp_ = 0.90, χ^2^ = 3.79, (*c*) *P*4_2_2_1_2, *a* = *b* = 125.69 (1) Å, *c* = 54.408 (7) Å, *R*_wp_ = 1.57, χ^2^ = 1.73, (*d*) *P*6_5_22, *a* = *b* = 58.01 (2) Å, *c* = 195.2 (7) Å, *R*_wp_ = 2.63, χ^2^ = 1.81.

Even when only a few peaks are present, accurate unit-cell parameters can be extracted from XRPD data sets using WPPF procedures (Karavassilia & Margiolaki, 2016 ▸; Margiolaki, 2019 ▸). On the basis of the starting lattice parameters, Pawley analysis (Pawley, 1981 ▸) theoretically simulates the experimental profiles in terms of peak shapes and background and, most importantly, allows for their refinement. Here, a structural model is not required, since peak intensities are considered as refinable parameters, contrary to Rietveld refinement (Rietveld, 1969 ▸). Using the reported unit-cell dimensions and space groups determined by X-ray crystallographic structure elucidation of *T. brucei* IMPDH and CatB (using *in cellulo* grown crystals), as well as of *P. pyralis* Luc and *N. crassa* HEX-1 (using crystals grown by microbatch and vapour diffusion techniques *in vitro*), as reasonable starting values (Supplementary Table S1), accurate lattice parameters were extracted for each data set (Fig. 5 ▸). A complete list of the refined reflections and their position in 2θ, *d* spacing and momentum transfer is presented in Supplementary Tables S2–S5.

For the Luc data set, the refined lattice parameters revealed a significant increase in the length of the unit-cell axes *a* and* b *by approximately 10 Å, compared with the expected values extracted from PDB entry 1lci (Conti *et al.*, 1996 ▸) (Supplementary Table S1). The unit cell of Luc *in cellulo* crystals has not been determined so far, but these differences indicate that the intracellular crystal growth affects the unit-cell geometry of Luc crystals. On the other hand, the detection of specific Bragg reflections from the intracellular Luc structures represents the first proof of the crystalline character. This result confirms our hypothesis that the intact cell protects the crystals from deterioration induced by environmental changes, *e.g.* during cell lysis and crystal isolation (Schönherr *et al.*, 2015 ▸).

For the other three data sets, Pawley analysis resulted in reasonable agreement of the refined and the expected unit-cell parameters (Supplementary Table S1). At least for IMPDH and CatB, this was expected, since the starting parameters have been obtained from the corresponding X-ray structures elucidated using these *in cellulo* grown crystals [IMPDH, PDB code 6rfu (Nass *et al.*, 2020 ▸); CatB, PDB 4hwy/4n4z (Redecke *et al.*, 2013 ▸; Gati *et al.*, 2014 ▸)]. However, the intracellular environment obviously did not change the unit-cell geometry of the HEX-1 crystals, as shown by the agreement with the parameters of crystals grown by applying the sitting drop vapour diffusion method (PDB code 1khi; Yuan *et al.*, 2003 ▸).

### 3.3. Sensitivity of *in cellulo* crystal detection   

**Figure 6:**
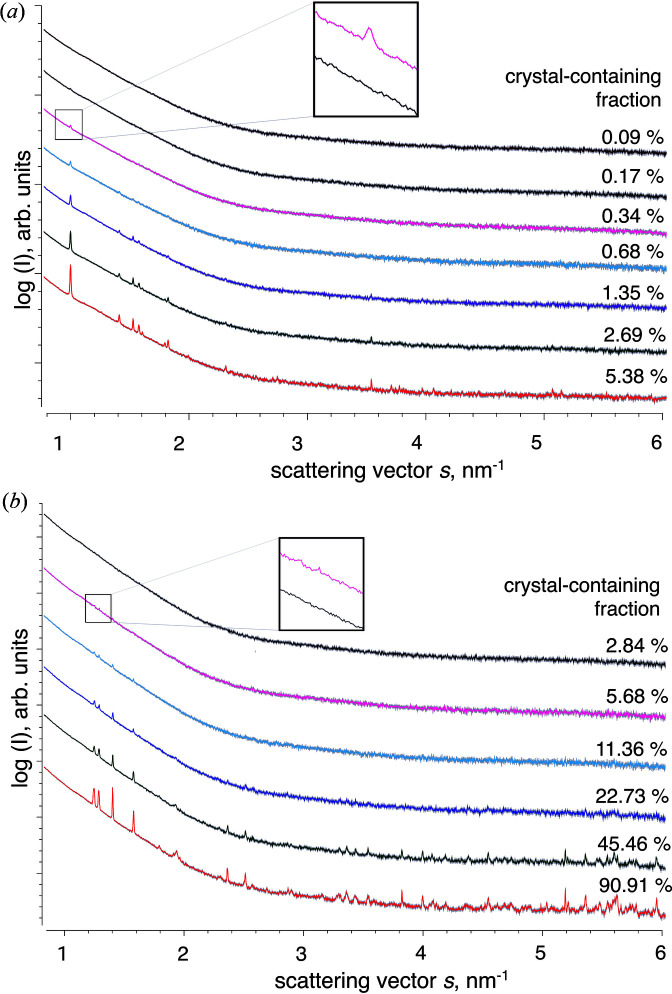
1D radially averaged X-ray scattering data of High Five insect cells containing intracellular crystals of the target proteins CatB (*a*) and HEX-1 (*b*) (SASBDB IDs: SASDH76 and SASDH66). The percentage of crystal-containing cells within the entire culture of each sample, as determined by light microscopy, is presented next to the scattering curves. The detection limit for *in cellulo* crystals using X-ray scattering at the P12 beamline setup 1 was determined to range between 0.3 and 6% of a crystal-containing cell fraction, depending on the respective protein. The insets show the scattering curve of the 16-fold diluted sample compared with that of mock-infected cells.

One of the major obstacles in intracellular protein crystallization is the observation that the proportion of crystal-containing cells within the entire culture can be very low. By applying light microscopy, one sometimes detects well ordered structures of a recombinant target protein in only 1% (or even less) of cells, rendering the proof of successful *in cellulo* crystallization a laborious and time-consuming effort. We have therefore further assessed the sensitivity of the scattering-based detection method by irradiation of a dilution series of High Five insect cells containing intracellular crystals of CatB and HEX-1. Starting from 100% infected cells, infected cells were diluted in a 1:2 ratio with mock-virus-infected cells. The intensity of the distinct diffraction peaks in the scattering curves consistently drops with each dilution step owing to the reduced number of crystals in the irradiated sample volume (Fig. 6 ▸). However, the overall course of the scattering curve was not affected. At a dilution of 16-fold, corresponding to 0.34 and 5.68% of cells in the sample that contain *in cellulo* CatB and HEX-1 crystals, respectively, even the originally most intense peaks can barely be distinguished from the background scattering from the cell suspensions. Progressive dilution yields scattering curves superimposable to that of the mock-virus-infected cells, defining the detection limit of the crystalline material in the irradiated volume at the specific conditions defined by this experimental setup. Considering the uncertainties in the determination of the detection limit, *e.g.* a slight volume increase of the insect cells after baculovirus infection (Schopf *et al.*, 1990 ▸) and individually varying cell sizes, this scattering approach enables the rapid detection of intracellular crystals of CatB and HEX-1 if present in at least 0.3–6% of all cells in the culture, depending on the individual protein. A comparable detection limit was determined for IMPDH *in cellulo* crystals in High Five cells (Supplementary Fig. S2).

### 3.4. Impact of the insect cell line   

**Figure 7:**
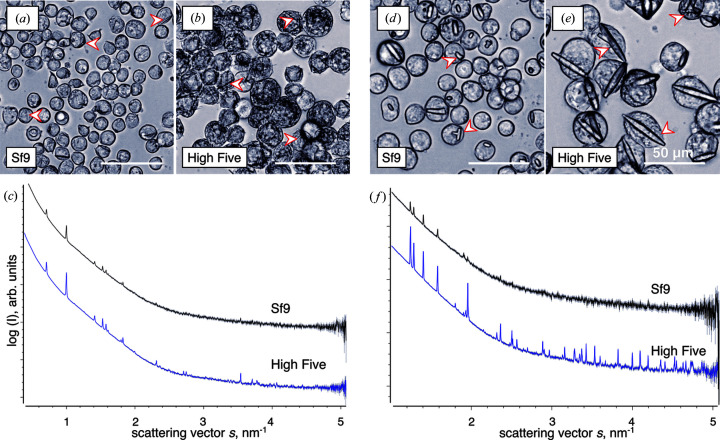
The unit-cell parameters of intracellular crystals do not depend on the insect cell line used for protein crystallization. Differential interference contrast light microscopy of intracellular CatB (*a*), (*b*) and HEX-1 (*d*), (*e*) crystals grown in Sf9 insect cells shows a highly comparable morphology compared with the growth in High Five cells. Red arrowheads highlight selected intracellular crystals. Corresponding X-ray scattering data show identical positions of the Bragg diffraction peaks in both cell lines for either CatB (SASBDB: SASDH96, SASDH86) (*c*) or HEX-1 (SASBDB: SASDHB6, SASDHA6) (*f*), indicating identical unit-cell parameters independent of the cell line used for crystal growth. The peak intensity is reduced in Sf9 cells owing to the observable drop in the crystallization efficiency. The standard deviation of each data point is presented as grey bars.

It was previously reported that the crystallization efficiency of recombinant target proteins in living insect cells varies depending on the individual cell line (Fan *et al.*, 1996 ▸). In High Five cell cultures, a larger proportion of cells produced intracellular crystals of the heterodimeric calcineurin complex, compared with Sf9 cell cultures. Our study clearly confirms this correlation. A significant drop in crystallization efficiency, ranging between 45 and 84%, was observed after infection of Sf9 cells with the same MOI of recombinant rBV stocks encoding CatB and HEX-1 (Fig. 7 ▸). Expectedly, the reduced crystalline scattering volume of the Sf9 cell samples leads to a decreased intensity of the distinct Bragg peaks in the scattering curves [Figs. 7 ▸(*c*) and 7(*f*)]. The peak positions, however, which directly depend on the symmetry and the unit-cell parameters of the irradiated crystals, did not change. Next to the important proof of the presence of crystalline material, the peak fingerprint obtained from the scattering data represents a precise and highly sensitive marker for the crystal architecture, which is not affected by the insect cell line, at least for the IMPDH, CatB and HEX-1 (Supplementary Fig. S2) proteins analysed in this study. This marker is much more reliable than the visual inspection of the crystals by light microscopy, which basically confirmed the needle-shaped tetragonal morphology of the IMPDH and CatB crystals and the elongated spindle-shaped hexagonal morphology of HEX-1 crystals, if grown in Sf9 cells (Fig. 7 ▸).

### 3.5. Timeline of intracellular crystal growth   

The timing of the X-ray measurements represents another parameter that essentially affects a reliable scoring of an *in cellulo* crystallization experiment. In the applied baculovirus expression vector system (BEVS; Smith *et al.*, 1983 ▸) recombinant target gene expression is controlled by the *Autographa californica* multiple nucleopolyhedrovirus (AcMNPV) polyhedrin promotor. Owing to its activation late in the infection cycle (Chambers *et al.*, 2018 ▸), target protein production starts approximately 24 h after rBV infection of the insect cells. First indications of intracellular crystal formation can be detected by light microscopy at least 72 h (3 days) p.i., as previously shown by real-time investigation of the spontaneous crystallization processes of *P. pyralis* Luc and GFP-μNS from avian reovirus (Schönherr *et al.*, 2015 ▸), as well as of *T. brucei* IMPDH (Nass *et al.*, 2020 ▸). Crystal growth usually continued up to day 5 p.i., when the majority of cells started to gradually lyse, triggered by the ongoing viral proliferation process. The associated environmental change can significantly affect the integrity and thus the X-ray diffraction capacity of *in cellulo* crystals (Schönherr *et al.*, 2015 ▸), defining the optimal time slot for intracellular crystal detection as between 24 and 120 h p.i. However, intracellular Luc crystals showed an unexpected dynamic degradation and reassembly within the same living cell over the entire growth period (Schönherr *et al.*, 2015 ▸), which turns the definition of the optimal time for detection into a more complicated task.

Samples with different offsets between the insect cell infection and the X-ray diffraction experiment have been prepared to monitor the time-dependent powder diffraction of High Five cells infected with rBVs encoding all four test proteins used in this study. On the basis of the results mentioned above, offsets ranging between 40 and 94.5 h were tested. In the scattering curves of cells producing Luc, CatB and HEX-1, Bragg diffraction peaks clearly distinguishable from the background scattering of the cells consistently appeared at approximately 51 h p.i. (Table 1 ▸ and Supplementary Fig. S4). Subsequently, the peak intensities increased up to approximately 81 h p.i. and remained constant. The Bragg peak intensities of IMPDH-producing cells exhibited a comparable trend, but the onset of crystal detection was delayed by 10 h, starting approximately at 61 h p.i. However, after 64 h p.i. the ratio of quantity, volume and intrinsic order of the crystalline material formed by all test proteins in the infected insect cells was consistently sufficient for detectable Bragg scattering, even if the single parameters were strongly dependent on the individual protein crystallization process. Our data indicate that the high brilliance and low background afforded by the SAXS instrument setup (*e.g.* all-in-vacuum beam path) enables a reliable scoring of *in cellulo* crystallization trials.

Obtaining insights into the kinetics of intracellular protein crystallization represents another reason to monitor the time dependence of *in cellulo* crystal growth. The associated molecular mechanisms are difficult to determine in the cellular context, preventing a comprehensive understanding so far. Initial insights have been obtained by live cell imaging techniques, but only after the size of the tracked crystal exceeded the detection limit of DIC light or fluorescence microscopy (Schönherr *et al.*, 2015 ▸), which is far beyond the nucleation event and the initial growth phase. Microscopy-based techniques are particularly problematic for proteins that exhibit a low *in cellulo* crystallization efficiency. These techniques focus on a few individual cells in the culture that have been selected by chance without guarantee that crystals will form inside these cells. Kinetic analysis by SONICC strongly depends on the orientation and symmetry of the growing crystals, which affects the signal intensity (Haupert *et al.*, 2012 ▸) and thus also the crystal detection. However, the cell selection problem is overcome by monitoring a large and representative fraction of all cells in the culture at the same time, as performed in X-ray powder-diffraction-based approaches. On the other hand, probing all cells at the same time without spatial resolution prevents the elucidation of the growth kinetics of a single crystal, since the total crystalline volume hit by the comparatively large X-ray beam contributes to the diffraction signal. Consequently, the low-background diffraction approach using high-brilliance X-ray beams will not provide more detailed insights into the crystallization process of individual crystals. However, it is able to provide information on the timing of the formation of detectable crystalline structures and a good estimate for overall crystal production inside the living cells, which is important to choose the optimal time point of further diffraction data collection at a synchrotron or XFEL for elucidation of the respective protein structure.

## 4. Conclusion   

Detection of intracellular crystals in cell cultures can be a time-consuming and challenging task, particularly if the target protein forms crystalline structures of unknown morphology only in a small fraction of cells. Furthermore, light-microscopy-based detection of well ordered structures yields a promising indication, but not a proof, of crystallinity. The presented SAXS–XRPD screening approach has the potential to overcome these major limitations of *in cellulo* crystallization. Owing to the automated robot-assisted sample handling, the flow-through setup, the short irradiation time and an exceptionally low background scattering of the SAXS beamline setup, this approach allows one within seconds to prove if diffracting crystalline structures of any order and morphology are present in at least a low percentage of cells within a culture. Such information cannot be obtained by other established detection methods in this time frame. Applying light microscopy, a comparable result would usually require several hours of tedious screening. Since the intensity of the X-rays determines the minimum diffractive volume that is required for reliable detection, a further increase in peak brilliance will allow the detection of smaller crystals or even a smaller percentage of crystal-containing cells, *e.g.* using fourth-generation synchrotrons or XFELs in the future. High-throughput SAXS–XRPD screening of potentially crystal-containing samples can be directly linked to subsequent serial diffraction data collection at a macromolecular crystallography beamline to streamline the structure determination. Moreover, since the Bragg peak positions in the 1D scattering curves depend on the unit-cell composition of the protein crystals, this approach also provides the possibility to investigate the impact of environmental conditions, *e.g.* the cellular compartment, cellular stress or the cell line itself, on the size and the composition of the intracellular protein crystals. This information could contribute to more detailed insights into the understanding of the *in cellulo* crystallization process.

## Supplementary Material

Supporting figures and tables. DOI: 10.1107/S1600576720010687/ei5059sup1.pdf

## Figures and Tables

**Table 1:** Timeline of intracellular crystal growth in High Five cells X-ray diffraction experiments using setup 2 have been performed at indicated time-points after insect cell infection with rBVs encoding Luc, IMPDH, CatB and HEX-1. The earliest time-point where a Bragg diffraction peak was detected is indicated with a plus sign (+). Arrows illustrate changes in the intensity of the Bragg peaks compared with the previous time-point. The consistent decrease of the signal intensity at the 64 h time-point is most likely attributable to a cell culture problem rather than to an effect of the intracellular crystallization process.

Protein	40 h	51.5 h	57.5 h	61.25 h	64 h	72.5 h	81.5 h	94.5 h
Luc	–	+	↗	↗	↘	↗	↗	→
IMPDH	–	–	–	+	↘	↗	↗	→
CatB	–	+	↗	↗	↘	↗	↗	→
HEX-1	–	+	→	→	↘	↗	↗	→
