# Supplementary material for: Rapid screening of in cellulo grown protein crystals via a small-angle X-ray scattering/X-ray powder diffraction synergistic approach
Source: J Appl Crystallogr. 2020 Sep 25;53(Pt 5):1169–80. doi: 10.1107/S1600576720010687 (PMC7534541; doi:10.1107/S1600576720010687)
Supplement: Supplementary file 1 [file j-53-01169-sup1.pdf]

JOURNAL OF APPLIED CRYSTALLOGRAPHY

Volume 53 (2020)

Supporting information for article:

Rapid screening of in cellulo grown protein crystals via a SAXS-XRPD synergistic approach

Janine Mia Lahey-Rudolph, Robert Schönherr, Cy M. Jeffries, Clément E. Blanchet, Juliane Boger, Ana Sofia Ferreira Ramos, Winnie Maria Riekehr, Dimitris-Panagiotis Triandafillidis, Alexandros Valmas, Irene Margiolaki, Dmitri Svergun and Lars Redecke

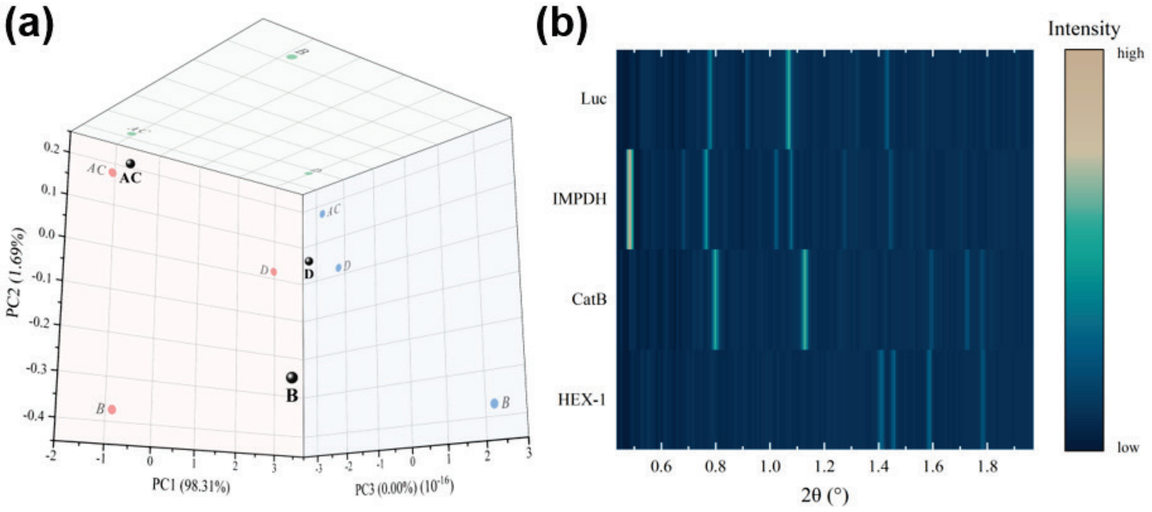

Figure S1 Results of principal component analysis of four synchrotron XRPD profiles based on the observed peak positions. **(a)** Four clusters were observed which correspond to A: Luc, B: IMPDH, C: CatB and D: HEX-1. **(b)** Diffraction intensity versus 2θ surface plot of the four XRPD datasets facilitate visual comparison of the individual peak positions. Brightly colored regions correspond to diffraction peaks and darker colored regions to background signal.

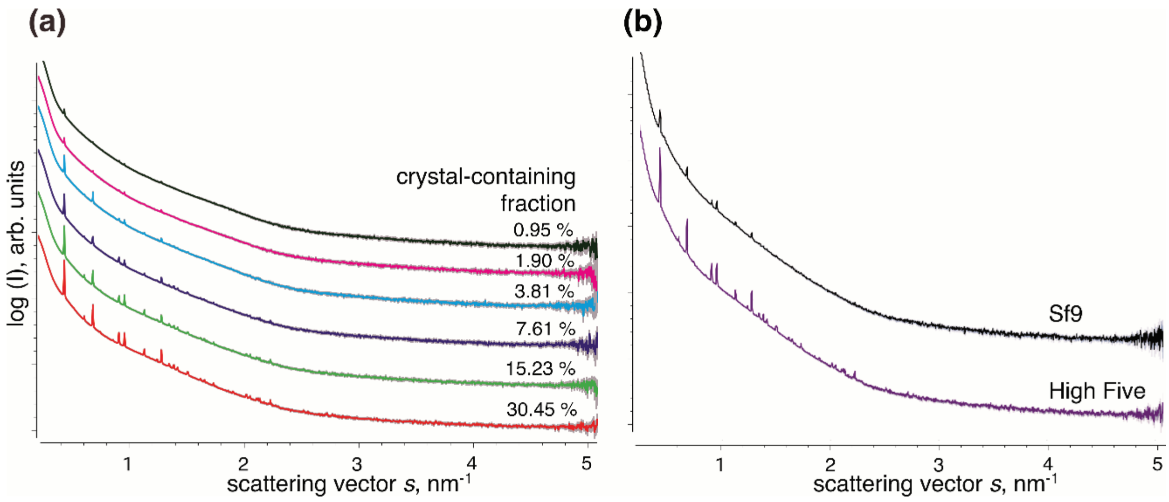

Figure S2 **(a)** 1D radially averaged X-ray scattering data of serial dilutions of High Five insect cells containing intracellular crystals of the target protein IMPDH**.** The percentage of crystal containing cells within the entire culture of each sample, as determined by light microscopy, is additionally presented next to the scattering curves. The apparent detection limit for IMPDH *in cellulo* crystals using X-ray scattering at the P12 beam line setup 2 was determined to be below 1 % of a crystal-containing cell fraction when diluted with uninfected High Five cells. The standard deviation of each data point is presented as grey bars. **(b)** The unit cell parameters of intracellular IMPDH crystals do not depend on the insect cell line used for protein crystallization. A reduced peak intensity is measured in Sf9 cells (SASBDB: SASDJZ5) compared to that in High Five cells (SASBDB: SASDJY5) due to the observable drop in the crystallization efficiency.

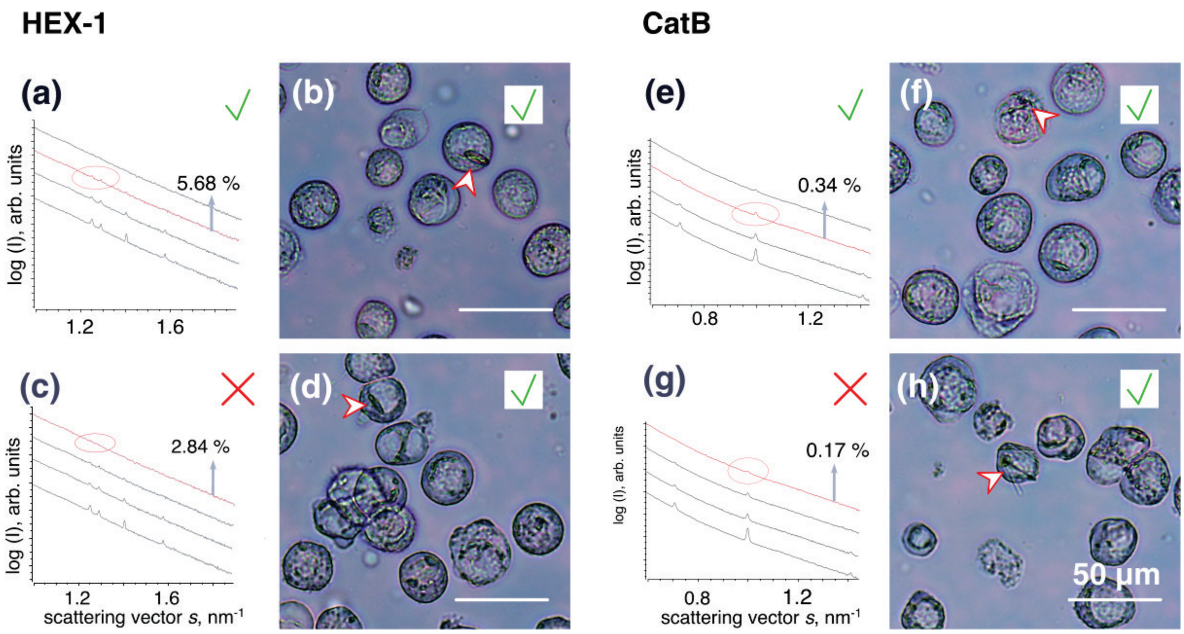

Figure S3 1D radially-averaged X-ray scattering data and light microscopy of baculovirus-infected High Five insect cells producing the recombinant proteins HEX-1 **(a-d)** and CatB **(d-h)** at dilution 1:16, corresponding to 5.68 % of HEX-1 **(a-b)** and to 0.34 % of CatB **(e-f)** crystal carrying cells, as well as at dilution 1:32, corresponding to 2.84 % and 0.17 % HEX-1 **(c-d)** and CatB **(g-h)** crystal carrying cells, respectively. Scattering curves are equivalent to Figure 5. The underlying grey bars on the scattering curves represent the standard deviation of each data point. Red arrowheads highlight selected intracellular crystals identified by light microscopy. Infected cells were examined on a Leica DM IL LED microscope in phase contrast mode. Images were taken 4 days after rBV infection in TBS directly before the diffraction experiment using a MC170HD camera.

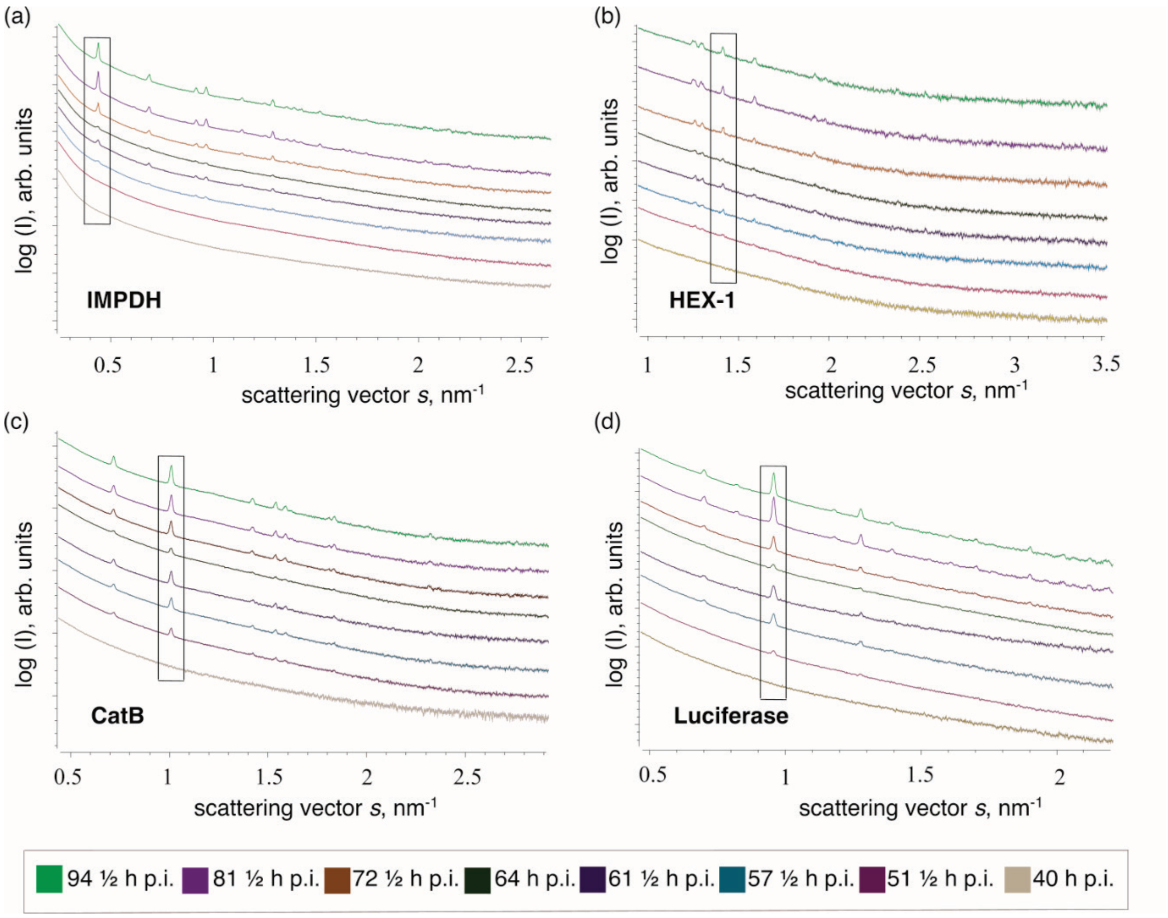

Figure S4 1D radially-averaged X-ray scattering data of baculovirus-infected High Five insect cells producing the recombinant proteins **(a)** IMPDH, **(b)** HEX-1, **(c)** CatB, and **(d)** luciferase, corresponding to defined time-points after infection of the cells as listed in table 1 and in the inset. Up to 40 hours after infection, no crystals can be detected. The strongest Bragg diffraction peak is highlighted with a black box and was used as a marker to estimate whether the diffraction intensity increased or decreased over time. The underlying grey bars on the scattering curves represent the standard deviation of each data point.

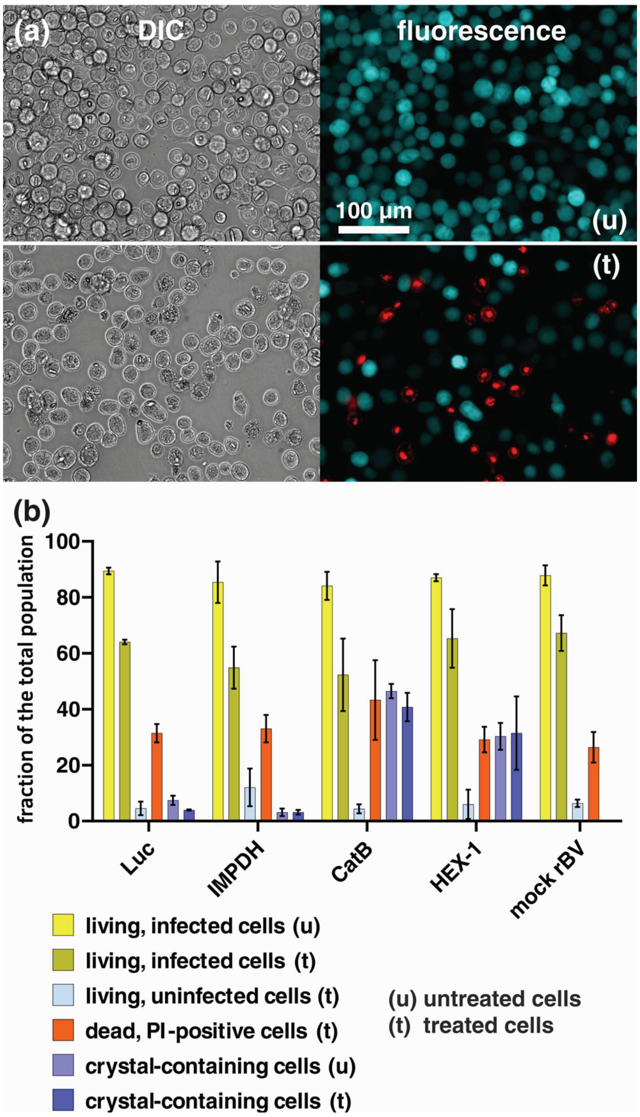

Figure S5 Sample preparation procedures did not affect the integrity of the predominant fraction of the infected Sf9 insect cells in the culture. **(a)** Cells imaged 4 days after infection with rBV HEX-1. *Upper panel:* Differential Interference Contrast light microscopy (DIC) and fluorescence microscopy of insect cells prior to sample preparation (u, untreated cells). EYFP fluorescence labelling of living, baculovirus-infected cells is shown in cyan. *Lower panel*: DIC and fluorescence microscopy of cells after sample preparation and propidium iodide (PI) staining (t, treated cells). PI fluorescence labelling of dead cells is shown in red. The scale bar applies to all panels. **(b)** Analysis of fractions of living, dead, uninfected, and crystal-containing cells prior to and after sample preparation procedures within different Sf9 cell cultures. More than 60 % of rBV-infected cells are still vital after sample preparation and thus at the beginning of the diffraction experiment. The reduction of luciferase crystal containing cells is due to the instability of luciferase crystals outside the living cell. rBV, recombinant baculovirus.

Table S1 Refined unit cell parameters as extracted from Pawley analysis of XRPD data. For every protein, unit cell parameters and space groups extracted from reported crystal structures listed in the PDB were used as starting values (estimated) for Pawley refinements. In addition to the refined unit cell dimensions the agreement factors of these fits are presented.

Protein	PDB code	Space group	Estimated		Refined		R_{wp}	X^2
			***a*, *b* (Å)**	***c* (Å)**	***a*, *b* (Å)**	***c* (Å)**		
P. pyralis Luc	1LCI	$P4_12_12$	119.53	94.68	129.13(6)	97.1(1)	0.80	1.76
T. brucei IMPDH	6RFU	$P42_12$	209.0	92.0	209.3(1)	93.44(2)	0.90	3.79
T. brucei CatB	3MOR	$P4_22_12$	125.4	54.6	125.69(1)	54.408(7)	1.57	1.73
N. crassa HEX-1	1KH1	$P6_522$	57.43	196.98	58.01(2)	195.2(7)	2.63	1.81

Table S2 List of the refined reflections in the Pawley analysis of XRPD data from the Luc *in cellulo* crystals grown in High Five cells (SASBDB: SASDHY5) and their position in 2θ, d-spacing and momentum transfer.

(h k l)	2θ (°)	d (Å)	s (nm^{-1})
(1 1 0)	0.7787	91.23515	0.68866
(1 0 1)	0.9164	77.53113	0.81043
(1 1 1)	1.069	66.46464	0.94538
(2 0 0)	1.101	64.52802	0.97368
(2 1 0)	1.2309	57.71904	1.08855
(2 0 1)	1.3223	53.73101	1.16938
(2 1 1)	1.4323	49.6061	1.26665
(2 2 0)	1.5569	45.63571	1.37684
(1 0 2)	1.5647	45.40744	1.38374
(1 1 2)	1.6587	42.83527	1.46686
(2 2 1)	1.7205	41.29669	1.52151
(3 1 0)	1.7406	40.81953	1.53928
(3 0 1)	1.8063	39.33349	1.59738
(2 0 2)	1.8322	38.77866	1.62028
(3 1 1)	1.8883	37.62607	1.66989
(2 1 2)	1.9131	37.13954	1.69182
(3 2 0)	1.9845	35.80267	1.75495
(3 2 1)	2.1153	33.58944	1.87061
(2 2 2)	2.1374	33.24195	1.89015
(4 0 0)	2.2016	32.27308	1.94692
(3 0 2)	2.2071	32.19205	1.95178
(1 0 3)	2.2647	31.373	2.00271
(4 1 0)	2.2693	31.30975	2.00678
(3 1 2)	2.2747	31.23575	2.01155
(4 0 1)	2.3201	30.62393	2.05169
(1 1 3)	2.3306	30.48589	2.06098
(3 3 0)	2.3351	30.42784	2.06496
(4 1 1)	2.3845	29.79722	2.10864
(3 3 1)	2.4472	29.03404	2.16407
(2 0 3)	2.4572	28.91632	2.17292
(4 2 0)	2.4614	28.86678	2.17663
(3 2 2)	2.4663	28.80875	2.18096
(2 1 3)	2.518	28.21731	2.22667

Table S3 List of the refined reflections in the Pawley analysis of XRPD data from the IMPDH *in cellulo* crystals grown in High Five cells (SASBDB: SASDHZ5) and their position in 2θ, d-spacing and momentum transfer.

(h k l)	2θ (°)	d (Å)	s (nm^{-1})
(1 1 0)	0.4861	146.167	0.42989
(2 0 0)	0.6849	103.7315	0.6057
(2 1 0)	0.765	92.86637	0.67654
(0 0 1)	0.7664	92.70473	0.67778
(1 0 1)	0.8387	84.70992	0.74172
(1 1 1)	0.9052	78.48416	0.80053
(2 2 0)	0.9661	73.53837	0.85438
(2 0 1)	1.0254	69.29005	0.90682
(3 1 0)	1.0795	65.81797	0.95467
(2 1 1)	1.0804	65.76048	0.95546
(3 2 0)	1.2299	57.76574	1.08767
(2 2 1)	1.2308	57.72689	1.08847
(3 0 1)	1.2769	55.63959	1.12923
(3 1 1)	1.3215	53.76377	1.16867
(4 0 0)	1.3638	52.09445	1.20608
(4 1 0)	1.4056	50.5457	1.24304
(3 3 0)	1.4462	49.12751	1.27895
(3 2 1)	1.4469	49.10362	1.27957
(4 2 0)	1.5241	46.6164	1.34783
(0 0 2)	1.5268	46.53494	1.35022
(4 0 1)	1.5623	45.4783	1.38161
(1 0 2)	1.5642	45.42154	1.38329

Table S4 List of the refined reflections in the Pawley analysis of XRPD data from CatB *in cellulo* crystals grown in High Five cells (SASBDB: SASDH26) and their position in 2θ, d-spacing and momentum transfer.

(h k l)	2θ (°)	d (Å)	s (nm^{-1})
(1 1 0)	0.7998	88.8338	0.70732
(2 0 0)	1.1309	62.82375	1.00012
(2 1 0)	1.2644	56.19327	1.11818
(1 0 1)	1.4233	49.91738	1.2587
(1 1 1)	1.5315	46.3918	1.35438
(2 2 0)	1.5992	44.42748	1.41424
(2 0 1)	1.7276	41.12513	1.52779
(3 1 0)	1.7879	39.73815	1.58111
(2 1 1)	1.8178	39.0857	1.60755
(3 2 0)	2.0385	34.85358	1.8027
(2 2 1)	2.0648	34.41086	1.82595
(3 0 1)	2.1408	33.1893	1.89316
(3 1 1)	2.2142	32.08923	1.95806
(4 0 0)	2.2615	31.41717	1.99988
(4 1 0)	2.3311	30.47928	2.06142
(3 3 0)	2.3987	29.62068	2.12119
(3 2 1)	2.4211	29.3475	2.141
(4 2 0)	2.5285	28.10087	2.23596
(4 0 1)	2.6116	27.20652	2.30943
(0 0 2)	2.6122	27.20006	2.30996
(4 1 1)	2.6721	26.59063	2.36292
(1 0 2)	2.6727	26.58459	2.36345
(3 3 1)	2.7313	26.01475	2.41526
(1 1 2)	2.7319	26.0091	2.41579
(4 3 0)	2.8269	25.13458	2.49978
(4 2 1)	2.8459	24.96703	2.51658
(2 0 2)	2.8465	24.96203	2.51711
(5 1 0)	2.8829	24.64655	2.54929
(2 1 2)	2.9021	24.48379	2.56626
(5 2 0)	3.0447	23.3371	2.69233
(2 2 2)	3.0629	23.19879	2.70842
(4 3 1)	3.1141	22.81722	2.75369
(5 0 1)	3.1141	22.81722	2.75369
(3 0 2)	3.1146	22.81341	2.75413
(5 1 1)	3.165	22.45025	2.79869
(3 1 2)	3.1655	22.44661	2.79913
(4 4 0)	3.1984	22.21639	2.82821
(5 3 0)	3.2968	21.55314	2.9152
(5 2 1)	3.3131	21.44721	2.92961
(3 2 2)	3.3136	21.44404	2.93005
(6 0 0)	3.3924	20.94596	2.99971
(6 1 0)	3.4392	20.66099	3.04108
(4 4 1)	3.4548	20.56763	3.05487
(4 0 2)	3.4553	20.56483	3.05531
(4 1 2)	3.5013	20.29495	3.09597
(5 3 1)	3.5462	20.03801	3.13566
(3 3 2)	3.5466	20.03542	3.13602
(6 2 0)	3.5759	19.87119	3.16192
(5 4 0)	3.6204	19.62739	3.20125
(6 0 1)	3.6352	19.5473	3.21433
(4 2 2)	3.6357	19.5449	3.21478
(6 1 1)	3.6789	19.31509	3.25296
(6 3 0)	3.7929	18.73485	3.35373
(6 2 1)	3.8071	18.66516	3.36628
(5 4 1)	3.8489	18.46267	3.40322
(4 3 2)	3.8493	18.46065	3.40358
(5 0 2)	3.8493	18.46065	3.40358
(5 1 2)	3.8906	18.26468	3.44008
(1 0 3)	3.9592	17.94839	3.50071
(5 5 0)	3.9981	17.77353	3.53509
(7 1 0)	3.9981	17.77353	3.53509
(1 1 3)	3.9994	17.76813	3.53624
(6 3 1)	4.0116	17.71399	3.54703
(5 2 2)	4.012	17.71221	3.54738
(6 4 0)	4.0774	17.42842	3.60518
(2 0 3)	4.0785	17.42332	3.60615
(7 2 0)	4.1164	17.26323	3.63965
(2 1 3)	4.1176	17.25827	3.64071
(4 4 2)	4.1298	17.20702	3.65149
(7 0 1)	4.168	17.04958	3.68525
(5 5 1)	4.2062	16.89484	3.71902
(7 1 1)	4.2062	16.89484	3.71902
(5 3 2)	4.2066	16.89329	3.71937
(2 2 3)	4.2325	16.78994	3.74226
(3 0 3)	4.2701	16.6421	3.77549
(6 4 1)	4.2815	16.59759	3.78556
(6 0 2)	4.2819	16.59613	3.78592
(7 3 0)	4.3062	16.50243	3.80739
(3 1 3)	4.3074	16.4981	3.80845
(7 2 1)	4.3187	16.45474	3.81844
(6 1 2)	4.3191	16.4533	3.81879
(6 5 0)	4.4163	16.09155	3.90469
(3 2 3)	4.4174	16.08753	3.90566
(6 2 2)	4.4288	16.04599	3.91574
(5 4 2)	4.4648	15.9168	3.94755
(7 3 1)	4.5001	15.79195	3.97874
(8 0 0)	4.5236	15.70991	3.99951
(4 0 3)	4.5247	15.70617	4.00048
(7 4 0)	4.5588	15.5886	4.03062
(8 1 0)	4.5588	15.5886	4.03062
(4 1 3)	4.5599	15.58495	4.03159
(3 3 3)	4.5948	15.4665	4.06243
(6 5 1)	4.6055	15.43076	4.07188
(6 3 2)	4.6058	15.42958	4.07215
(8 2 0)	4.6629	15.24089	4.12261
(4 2 3)	4.6639	15.23748	4.12349
(8 0 1)	4.7085	15.09327	4.1629
(7 4 1)	4.7424	14.9856	4.19285
(8 1 1)	4.7424	14.9856	4.19285
(7 0 2)	4.7427	14.98452	4.19312
(5 5 2)	4.7763	14.87915	4.22281
(7 1 2)	4.7763	14.87915	4.22281
(6 6 0)	4.7981	14.81151	4.24207
(8 3 0)	4.8313	14.70972	4.27141
(4 3 3)	4.8324	14.70666	4.27238
(5 0 3)	4.8324	14.70666	4.27238
(8 2 1)	4.8425	14.67592	4.2813
(6 4 2)	4.8428	14.6749	4.28157
(7 5 0)	4.8643	14.61	4.30056
(5 1 3)	4.8653	14.607	4.30145
(7 2 2)	4.8757	14.57588	4.31064
(5 2 3)	4.963	14.31976	4.38777
(6 6 1)	4.9729	14.29138	4.39652

Table S5 List of the refined reflections in the Pawley analysis of the *N. crassa* HEX-1 *in cellulo* crystals XRPD data (SASBDB: SASDH36) and their position in 2θ, d-spacing and momentum transfer.

(h k l)	2θ (°)	d (Å)	s (nm^{-1})
(1 0 0)	1.4106	50.36847	1.24747
(1 0 1)	1.4567	48.77536	1.28823
(1 0 2)	1.5869	44.77252	1.40337
(1 0 3)	1.783	39.84872	1.57677
(1 0 4)	2.0259	35.07033	1.79156
(0 0 6)	2.1799	32.59321	1.92773
(1 0 5)	2.301	30.87858	2.03481
(1 1 0)	2.446	29.04838	2.16301
(1 1 1)	2.4729	28.73254	2.1868
(1 1 2)	2.5519	27.84344	2.25665
(1 0 6)	2.598	27.34906	2.2974
(1 1 3)	2.6783	26.52891	2.3684
(2 0 0)	2.825	25.15159	2.4981
(1 1 4)	2.846	24.96645	2.51667
(2 0 1)	2.8484	24.94569	2.51879
(1 0 7)	2.9103	24.41518	2.57351
(2 0 2)	2.9172	24.35706	2.57961
(2 0 3)	3.0285	23.46227	2.67801
(1 1 5)	3.048	23.31176	2.69525
(2 0 4)	3.1777	22.36046	2.80991
(1 0 8)	3.2334	21.97594	2.85915
(1 1 6)	3.2782	21.67571	2.89876
(2 0 5)	3.36	21.14821	2.97107
(1 1 7)	3.5309	20.12474	3.12214
(1 0 9)	3.5644	19.9356	3.15175
(2 0 6)	3.5701	19.90391	3.15679
(2 1 0)	3.7386	19.0068	3.30573
(2 1 1)	3.7563	18.91744	3.32138
(1 1 8)	3.8017	18.69157	3.3615
(2 0 7)	3.8035	18.68285	3.3631
(2 1 2)	3.8088	18.65676	3.36778
(2 1 3)	3.8947	18.24525	3.44371
(1 0 10)	3.9013	18.21443	3.44954
(2 1 4)	4.012	17.71234	3.54738
(2 0 8)	4.0562	17.51933	3.58645
(1 1 9)	4.087	17.38708	3.61367
(2 1 5)	4.1578	17.09114	3.67624
(3 0 0)	4.2399	16.76048	3.7488
(1 0 11)	4.2428	16.74912	3.75136
(3 0 1)	4.2555	16.69911	3.76258
(3 0 2)	4.3019	16.51896	3.80359
(2 0 9)	4.3248	16.43161	3.82383
(2 1 6)	4.3295	16.41386	3.82798
(0 0 12)	4.3643	16.28293	3.85874
(3 0 3)	4.3782	16.23125	3.87102
(1 1 10)	4.3841	16.20953	3.87623
(3 0 4)	4.4829	15.85258	3.96355
(2 1 7)	4.524	15.70856	3.99986
(1 0 12)	4.5878	15.49024	4.05624
(2 0 10)	4.6066	15.42701	4.07286
(3 0 5)	4.6139	15.40253	4.07931
(1 1 11)	4.6906	15.15076	4.14708
(2 1 8)	4.7385	14.99776	4.18941
(3 0 6)	4.7692	14.90124	4.21653
(2 2 0)	4.8968	14.51332	4.32928
(2 0 11)	4.8993	14.50594	4.33149
(2 2 1)	4.9103	14.47341	4.34121
(1 0 13)	4.9356	14.39933	4.36356
(3 0 7)	4.9465	14.36751	4.37319
(2 2 2)	4.9506	14.35564	4.37682
(2 1 9)	4.9705	14.29819	4.3944
(1 1 12)	5.0049	14.19989	4.42479
(2 2 3)	5.0171	14.16557	4.43557
(3 1 0)	5.097	13.94354	4.50616
(2 2 4)	5.1087	13.91173	4.5165
(3 1 1)	5.11	13.90814	4.51765
(3 0 8)	5.1435	13.81761	4.54725
(3 1 2)	5.1487	13.80353	4.55184
(2 0 12)	5.201	13.66489	4.59805
(3 1 3)	5.2127	13.6343	4.60838
(2 1 10)	5.2176	13.62142	4.61271
(2 2 5)	5.2241	13.60456	4.61845
(1 0 14)	5.2856	13.44634	4.67279
(3 1 4)	5.3009	13.40751	4.6863
(1 1 13)	5.3256	13.34538	4.70812
(3 0 9)	5.358	13.26474	4.73674
(2 2 6)	5.3618	13.25539	4.7401
(3 1 5)	5.4123	13.1319	4.78471
(2 1 11)	5.4778	12.9748	4.84257
(2 0 13)	5.5103	12.89834	4.87128
(2 2 7)	5.5201	12.87545	4.87994
(3 1 6)	5.5453	12.81704	4.9022
(3 0 10)	5.5881	12.71902	4.94001
(1 0 15)	5.6375	12.60763	4.98364
(1 1 14)	5.6516	12.57612	4.9961
(4 0 0)	5.6554	12.56764	4.99945
(4 0 1)	5.6672	12.5417	5.00988
(2 2 8)	5.6974	12.4752	5.03655
(3 1 7)	5.6986	12.4726	5.03761
(4 0 2)	5.7021	12.46483	5.0407
(2 1 12)	5.7494	12.36245	5.08248
(4 0 3)	5.76	12.33979	5.09184
(2 0 14)	5.8261	12.19991	5.15023
(3 0 11)	5.8319	12.18779	5.15535
(4 0 4)	5.84	12.17089	5.1625
(3 1 8)	5.8705	12.10771	5.18944
(2 2 9)	5.8919	12.06378	5.20834
(4 0 5)	5.9412	11.96358	5.25188
(1 1 15)	5.9821	11.88194	5.28801
(1 0 16)	5.9909	11.86447	5.29578
(2 1 13)	6.0308	11.78617	5.33102
(3 1 9)	6.0594	11.73051	5.35627
(4 0 6)	6.0627	11.72404	5.35919
(3 0 12)	6.0877	11.67603	5.38127
(2 2 10)	6.1019	11.64888	5.39381
(2 0 15)	6.1472	11.56312	5.43381
(3 2 0)	6.1637	11.53224	5.44838
(3 2 1)	6.1744	11.51219	5.45783
(4 0 7)	6.2033	11.45868	5.48335
(3 2 2)	6.2066	11.45265	5.48627
(3 2 3)	6.2597	11.35543	5.53316
(3 1 10)	6.2639	11.34798	5.53687
(1 1 16)	6.3164	11.25375	5.58323
(2 1 14)	6.3206	11.24614	5.58694
(2 2 11)	6.326	11.23664	5.5917
(3 2 4)	6.3335	11.22339	5.59833
(1 0 17)	6.3456	11.20186	5.60901
(3 0 13)	6.3542	11.18686	5.6166

(h k l)	2θ (°)	d (Å)	s (nm^{-1})
(4 0 8)	6.3616	11.17378	5.62314
(3 2 5)	6.427	11.0602	5.68089
(2 0 16)	6.473	10.98174	5.7215
(4 1 0)	6.4805	10.96903	5.72813
(3 1 11)	6.4824	10.96585	5.7298
(4 1 1)	6.4907	10.95177	5.73713
(4 1 2)	6.5213	10.90047	5.76415
(4 0 9)	6.5364	10.87525	5.77748
(3 2 6)	6.5395	10.8701	5.78022
(0 0 18)	6.5503	10.85224	5.78976
(2 2 12)	6.5627	10.8318	5.8007
(4 1 3)	6.5719	10.81654	5.80883
(2 1 15)	6.6179	10.74146	5.84944
(3 0 14)	6.63	10.72195	5.86012
(4 1 4)	6.6422	10.70224	5.87089
(1 1 17)	6.6538	10.68357	5.88114
(3 2 7)	6.6701	10.65758	5.89553
(1 0 18)	6.7015	10.60771	5.92325
(3 1 12)	6.7136	10.58861	5.93393
(4 0 10)	6.7265	10.56834	5.94532
(4 1 5)	6.7315	10.56046	5.94973
(2 0 17)	6.8027	10.45001	6.01259
(2 2 13)	6.8107	10.43783	6.01965
(3 2 8)	6.8176	10.4272	6.02575
(4 1 6)	6.839	10.3946	6.04464
(3 0 15)	6.9141	10.28194	6.11093
(2 1 16)	6.9217	10.27062	6.11764
(4 0 11)	6.9305	10.25761	6.12541
(3 1 13)	6.9562	10.21969	6.1481
(4 1 7)	6.964	10.2083	6.15498
(3 2 9)	6.9811	10.18334	6.17008
(1 1 18)	6.9941	10.16444	6.18155
(1 0 19)	7.0583	10.07214	6.23822
(2 2 14)	7.0688	10.05715	6.24749
(5 0 0)	7.0718	10.05281	6.25014
(5 0 1)	7.0812	10.03952	6.25844
(4 1 8)	7.1055	10.0053	6.27989
(5 0 2)	7.1093	9.99995	6.28324
(2 0 18)	7.1359	9.96268	6.30672
(4 0 12)	7.1473	9.94685	6.31678
(5 0 3)	7.1558	9.93503	6.32429
(3 2 10)	7.1594	9.93004	6.32746
(3 0 16)	7.2054	9.86671	6.36806
(3 1 14)	7.2092	9.86158	6.37142
(5 0 4)	7.2204	9.84623	6.3813
(2 1 17)	7.2311	9.83169	6.39075
(4 1 9)	7.2625	9.78926	6.41846
(5 0 5)	7.3027	9.73549	6.45394
(2 2 15)	7.336	9.69133	6.48333
(1 1 19)	7.3367	9.69035	6.48395

(h k l)	2θ (°)	d (Å)	s (nm^{-1})
(3 3 0)	7.3498	9.67313	6.49551
(3 2 11)	7.3515	9.67094	6.49701
(3 3 1)	7.3588	9.66129	6.50345
(4 0 13)	7.3757	9.63915	6.51837
(3 3 2)	7.3858	9.62601	6.52728
(5 0 6)	7.4019	9.60507	6.54149
(1 0 20)	7.4159	9.58708	6.55385
(3 3 3)	7.4306	9.56806	6.56682
(4 1 10)	7.4341	9.56361	6.56991
(3 1 15)	7.4713	9.51597	6.60274
(2 0 19)	7.4721	9.51504	6.60344
(4 2 0)	7.4849	9.49874	6.61474
(3 3 4)	7.4929	9.48867	6.6218
(4 2 1)	7.4938	9.48752	6.62259
(3 0 17)	7.5032	9.47565	6.63089
(5 0 7)	7.5176	9.4575	6.6436
(4 2 2)	7.5203	9.45411	6.64598
(2 1 18)	7.5455	9.42259	6.66822
(3 2 12)	7.5563	9.4092	6.67775
(4 2 3)	7.5643	9.39919	6.68481
(3 3 5)	7.5722	9.38943	6.69178
(2 2 16)	7.6113	9.34128	6.72628
(4 0 14)	7.6148	9.33693	6.72937
(4 1 11)	7.6193	9.33149	6.73334
(4 2 4)	7.6255	9.32389	6.73881
(5 0 8)	7.6489	9.29539	6.75946
(3 3 6)	7.668	9.27227	6.77631
(1 1 20)	7.6814	9.25608	6.78814
(4 2 5)	7.7034	9.22969	6.80755
(3 1 16)	7.7419	9.18394	6.84152
(3 2 13)	7.7728	9.14748	6.86879
(1 0 21)	7.7742	9.14582	6.87002
(3 3 7)	7.7797	9.1393	6.87487
(5 0 9)	7.7951	9.12138	6.88846
(4 2 6)	7.7977	9.11833	6.89076
(3 0 18)	7.8067	9.10779	6.8987
(2 0 20)	7.8109	9.10294	6.9024
(4 1 12)	7.8171	9.09569	6.90787
(4 0 15)	7.8636	9.042	6.9489
(2 1 19)	7.8643	9.0412	6.94952
(5 1 0)	7.8765	9.02721	6.96028
(5 1 1)	7.8849	9.01758	6.96769
(2 2 17)	7.8939	9.00738	6.97563
(3 3 8)	7.9067	8.99276	6.98693
(4 2 7)	7.9076	8.99179	6.98772
(5 1 2)	7.9101	8.98887	6.98993
(5 1 3)	7.952	8.94163	7.02689
(5 0 10)	7.9552	8.938	7.02972
(3 2 14)	8.0001	8.88797	7.06933
